# A computational method for sharp interface advection

**DOI:** 10.1098/rsos.160405

**Published:** 2016-11-23

**Authors:** Johan Roenby, Henrik Bredmose, Hrvoje Jasak

**Affiliations:** 1 DHI, Department of Ports and Offshore Technology, Agern Allé 5, 2970 Hørsholm, Denmark; 2 Department of Wind Energy, Technical University of Denmark, 2800 Kgs. Lyngby, Denmark; 3 University of Zagreb, Faculty of Mechanical Engineering and Naval Architecture, Ivana Lučića 5, Zagreb, Croatia; 4 Wikki Ltd, 459 Southbank House, SE1 7SJ, London, UK

**Keywords:** interfacial flows, volume of fluid method, unstructured meshes, isoAdvector, OpenFOAM^®^

## Abstract

We devise a numerical method for passive advection of a surface, such as the interface between two incompressible fluids, across a computational mesh. The method is called isoAdvector, and is developed for general meshes consisting of arbitrary polyhedral cells. The algorithm is based on the volume of fluid (VOF) idea of calculating the volume of one of the fluids transported across the mesh faces during a time step. The novelty of the isoAdvector concept consists of two parts. First, we exploit an isosurface concept for modelling the interface inside cells in a geometric surface reconstruction step. Second, from the reconstructed surface, we model the motion of the face–interface intersection line for a general polygonal face to obtain the time evolution *within* a time step of the submerged face area. Integrating this submerged area over the time step leads to an accurate estimate for the total volume of fluid transported across the face. The method was tested on simple two-dimensional and three-dimensional interface advection problems on both structured and unstructured meshes. The results are very satisfactory in terms of volume conservation, boundedness, surface sharpness and efficiency. The isoAdvector method was implemented as an OpenFOAM^®^ extension and is published as open source.

## 1. Introduction

In this paper, we address the numerical challenge of advancing a surface moving in a prescribed velocity field. We will refer to this as the interface advection problem, because the surface often constitutes an interface e.g. between two fluids. Simple as the problem may appear, there is a large variety of problems in science, engineering and industry where its solutions are far from trivial. Our motivation for addressing this problem is rooted in our usage of computational fluid dynamics (CFD) as a practical engineering tool for calculating wave loads on coastal and marine structures. Whether it is an offshore wind turbine foundation, or an oil and gas platform, accurate estimation of the peak loads from violent breaking waves is paramount for the correct dimensioning of the structure. In our view, CFD has a large unexploited potential to improve wave load estimates, and to reduce both cost and risks in the design phase of coastal and offshore structures.

Owing to the omnipresence of interfacial flows, the list of areas that may benefit from improved solution methods to the interface advection problem is almost endless. Some examples are bubble column reactors, oil–gas mixtures in pipelines, inkjet printing, automotive aquaplaning, ship manoeuvring, tank sloshing, dam breaks, metal casting processes and hydraulic jumps.

During the past 40–50 years, both Lagrangian and Eulerian strategies have been employed to develop a wide range of methods for advecting a sharp interface [1]. Today, most CFD codes for practical engineering calculations use variants of the *volume-of-fluid (VOF)* method for the interface advection step in their interfacial flow solvers. This includes current versions of ANSYS Fluent^®^, STAR-CCM+^®^, Gerris [2], OpenFOAM^®^ [3,4] and many others. In the VOF methodology, the interface is implicitly represented via the *volume fractions* of one of the fluids in computational cells. The advection is done by redistributing the content of this fluid between adjacent cells by moving it across the mesh faces. Since the first VOF methods appeared in the literature [5], a large variety of VOF schemes have been developed. They may be divided into two categories: *geometric* methods involving an explicit reconstruction of the interface from the volume fraction data, and *algebraic* methods making no such attempt. Algebraic VOF schemes are typically much simpler to implement, more efficient and are not restricted to structured meshes. They are, however, founded on much more heuristic considerations and are not as accurate as the geometric VOF schemes [6]. Geometric VOF schemes, on the other hand, involve complex geometric operations making their implementation cumbersome and their execution slow. Geometric VOF methods for unstructured meshes is an active area of research [7–13].

Our ambition in the development of the isoAdvector algorithm is to develop a VOF-based interface advection method that works on arbitrary meshes, retains the accuracy of the geometric schemes by explicitly approximating the interface, and yet keeps the geometric operations at a minimum in order to obtain acceptable calculation times. An efficient VOF scheme yielding accurate results even on automatically generated unstructured meshes of complex geometries has a huge potential for speeding up the simulation process and making CFD an integrated part of design processes involving interfacial flows.

In the remainder of this section, we give an introduction to the interface advection problem and its formulation in the VOF framework. In §2, we present the new ideas and concepts of the isoAdvector method, and give an overview of the steps involved in the numerical procedure. The implementation details and considerations involved in each step are described at length in §3. In §4, we demonstrate the performance of the new method with a series of simple test cases. Finally, in §5 we summarize our findings.

### 1.1. VOF formulation of the interface advection problem

We consider a computational domain $D\in R^{3}$ in which a surface S is embedded. The surface may consist of any number of closed surfaces and may also extend to the boundaries of the domain. We will think of the surface, S, as the interface between two incompressible, immiscible fluids denoted by A and B, and occupying the two closed regions, A and B, satisfying A∩B=S and A∪B=D.

The fluid particles are assumed to be passively advected in a continuous, solenoidal velocity field, **u**(**x**,*t*), which is defined in the whole domain, D. In practical engineering applications involving incompressible two-phase flows, the time evolution of the velocity field is governed by the Navier–Stokes equations for **u** coupled with a Poisson equation for the pressure, *p*. This system of equations must be solved simultaneously with the interface advection problem. In this work, we focus entirely on the interface advection problem, thus assuming **u**(**x**,*t*) to be known in advance for all points, x∈D, and all times, *t*.

We will now represent the surface S(t) in terms of a density field, *ρ*(**x**,*t*), which takes one constant value, *ρ*_*A*_, everywhere in A and another constant value, *ρ*_*B*_, everywhere in B. The density field thus has a discontinuity at the interface S.^1^ The evolution of the surface is then determined by the integral form of the continuity equation,

1.1 $$\frac{d}{dt}\int_{V}\rho (\text{x},t)\;dV=-\int_{\partial V}\rho (\text{x},t)\text{u}(\text{x},t)\cdot d\text{S},$$

where V∈D is an arbitrary stationary volume, ∂V is its boundary and *d***S** is the differential area vector pointing out of the volume. In words, this mass conservation equation says that the instantaneous rate of change of the total mass enclosed in the volume is given by the instantaneous flux of mass through its boundary.

In the pure advection problem with a predetermined velocity field, the specific values of the fluid densities, *ρ*_*A*_ and *ρ*_*B*_, are immaterial, that is, the solution does not depend on them. To remove these insignificant parameters from the problem, we define the indicator field,

1.2 $$H(\text{x},t)\equiv \frac{\rho (\text{x},t)-\rho_{B}}{\rho_{A}-\rho_{B}},$$

such that *H*=1 for all x∈A(t), and *H*=0 for all x∈B(t).

We now discretize the computational domain, D, by conceptually dividing it into a large number of control volumes, or *cells*, $C_{i}$, for *i*=1,…,*N*_*C*_. If two cells *i* and *j* are adjacent, their shared boundary, $\partial C_{i}\cap \partial C_{j}$, is called an *internal face*. If cell *i* touches the domain boundary, the shared surface, $\partial C_{i}\cap \partial D$, will consist of one or more *boundary faces*. All faces are labelled with integers, *j*=1,…,*N*_*F*_, and the surface of face *j* is denoted by $F_{j}$. Thus, the boundary of the cell *i* may be represented by a list, *B*_*i*_, of all the labels of faces belonging to its boundary $\partial C_{i}$.

With these mesh definitions in place, we can now substitute (1.2) into (1.1) with cell *i* as the volume of integration,

1.3 $$\frac{d}{dt}\int_{C_{i}}H(\text{x},t)\;dV=-\sum_{j\in B_{i}}s_{ij}\int_{F_{j}}H(\text{x},t)\text{u}(\text{x},t)\cdot d\text{S}.$$

Because face *j* has its own orientation determining the direction of *d***S**, we have introduced the auxiliary factor *s*_*ij*_=+1 or −1, such that *s*_*ij*_ *d***S** points out of cell *i* for face *j*.

The natural next step is to define the volume fraction of fluid A in cell *i*,

1.4 $$\alpha_{i}(t)\equiv \frac{1}{V_{i}}\int_{C_{i}}H(\text{x},t)\;dV,$$

where *V*_*i*_ is the volume of cell *i*. Substituting (1.4) into (1.3), and formally integrating (1.3) from time *t* to time *t*+Δ*t*, we obtain the following equation for the updated volume fraction of cell *i*,

1.5 $$\alpha_{i}(t+\Delta t)=\alpha_{i}(t)-\frac{1}{V_{i}}\sum_{j\in B_{i}}s_{ij}\int_{t}^{t+\Delta t}\int_{F_{j}}H(\text{x},\tau )\text{u}(\text{x},\tau )\cdot d\text{S}\;d\tau .$$

We stress that this equation is exact with no numerical approximations introduced yet. It is the fundamental equation from which one must derive any consistent interface advection method. The time integral on the right-hand side is the total volume of fluid A transported across face *j* during the time interval from time *t* to *t*+Δ*t*. It is the fundamental quantity that we must estimate in order to advance *α*_*i*_, and hence implicitly the surface S, in time. We will call this quantity Δ*V*_*j*_(*t*,Δ*t*):

1.6 $$\Delta V_{j}(t,\Delta t)\equiv \int_{t}^{t+\Delta t}\int_{F_{j}}H(\text{x},\tau )\text{u}(\text{x},\tau )\cdot d\text{S}\;d\tau .$$

The fundamental equation (1.5) can then be formulated as

1.7 $$\alpha_{i}(t+\Delta t)=\alpha_{i}(t)-\frac{1}{V_{i}}\sum_{j\in B_{i}}s_{ij}\Delta V_{j}(t,\Delta t).$$

Before we move on to present the basic ideas of the isoAdvector method, we will need to consider how the velocity field is represented. In the finite volume treatment of the fluid equations of motion, the natural representation of the velocity field is in terms of cell-averaged values,

1.8 $${\text{u}}_{i}(t)\equiv \frac{1}{V_{i}}\int_{C_{i}}\text{u}(\text{x},t)\;dV.$$

As the convective terms in the governing fluid equations give the transport of mass, momentum, etc. across cell faces, other important velocity field representations are the volumetric fluxes across mesh faces,

1.9 $$\phi_{j}(t)\equiv \int_{F_{j}}\text{u}(\text{x},t)\cdot d\text{S}.$$

The question we will try to answer in the following can now be formulated as follows:

How do we most accurately and efficiently exploit the available information at time *t*, i.e. the volume fractions, *α*_*i*_, and the velocity data, *u*_*i*_ and *ϕ*_*j*_, to estimate the fluid A volume transport, Δ*V*_*j*_(*t*,Δ*t*), across a face during the time interval [*t*,*t*+Δ*t*]?

## 2. The isoAdvector concept

We will now present the general ideas behind the isoAdvector method, starting with the interface representation using isosurfaces, then introducing the concept of a *face–interface intersection line* moving across a face, and finally giving an overview of the steps involved in the numerical procedure. For the sake of clarity, we focus on ideas in this section, and postpone the detailed description of the implementation to §3. For full implementation details, the reader is referred to the source code provided with this article [14].

### 2.1. The interface reconstruction step

The integral in (1.6) is highly dependent on the local distribution of fluid A and B inside cell *i* and inside its neighbour cells from which it receives fluid during the time step. However, the volume fractions hold no information about the distribution of the two fluids inside the cells. We must therefore come up with a subgrid model for this ‘intracellular’ distribution from the given volume fraction data. If the volume fraction data are ‘sharp’, only cells very close to the interface will have volume fractions significantly different from 0 and 1. Then, if cell *i* is on the interface, its neighbours in one direction will mainly contain fluid A, while its neighbour cells in the opposite direction will mainly contain fluid B. In words, we want our subgrid model to capture this local distribution information, and place the fluid A content of cell *i* close to the neighbours containing fluid A (which is equivalent to its fluid B content being placed near the neighbours containing fluid B). The implicit assumption made in this model is that the interface is sufficiently well resolved by the mesh such that the local radius of curvature is larger than the cell size. Whenever this is satisfied, an isosurface calculation will provide a good estimate of the required local fluid distribution information.

**Figure 1.**
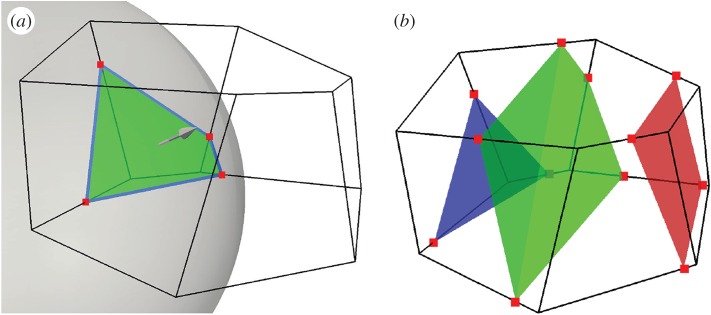
(*a*) A spherical surface cutting a polyhedral cell. Red dots are the edge cutting points. Blue lines are the face–interface intersection lines. Green patch is the isoface. (*b*) The isoface motion is estimated from surrounding velocity data and the isoface is propagated. Isoface at three different times within a time step are shown.

The idea of using isosurface calculations in the interface reconstruction step is inspired by our work with postprocessing of interfacial CFD data. Here, it is customary to visualize the fluid interface (e.g. using ParaView^®^ [15]) by showing the 0.5-isosurface based on the volume fraction data. Such numerically calculated isosurfaces are topologically consistent continuous surfaces and are straightforward to calculate on arbitrary polyhedral meshes. The numerical representation of an isosurface in a polyhedral cell is a list of the points where the isosurface cuts the cell edges. See red points in figure 1*a* for an illustration. This list of points represents a face which cuts the cell into two polyhedral subcells, with one completely immersed in fluid A and the other completely immersed in fluid B. We will call such a face an *isoface*. See the green patch in figure 1*a* for an example. We note that if an isoface has more than three vertices, it will generally not be exactly planar.

When calculating an isosurface from the volume fraction data, we have the freedom of choosing an isovalue between 0 and 1. Which isovalue should we choose? For surface visualization from volume fraction data, we usually plot the 0.5-isosurface. This, however, is not a good choice for the surface reconstruction step in an interface advection algorithm, because the isoface in cell *i* with isovalue 0.5 does not in general cut it into two subcells of the volumetric proportions dictated by the volume fraction, *α*_*i*_. It may, for instance, occur that the cell has *α*_*i*_=0.8, and yet is not even cut by the 0.5-isosurface. In this case, a surface reconstruction model based on the 0.5-isosurface would say nothing about how the 80% fluid A and 20% fluid B is distributed inside this cell. There will, however, exist an isoface with another isovalue, which will cut the cell into subcells of the correct volumetric proportions. An important component of our proposed scheme is an efficient method for finding this isovalue for a given surface cell (see §3 for details). We note that with the use of different isovalues in different surface cells, the union of isofaces is no longer a continuous surface, as it would be if the same isovalue was used in adjacent cells. This is the price we must pay to ensure that isofaces cut surface cells into subcells having the correct volumetric proportions.

### 2.2. The advection step

Most Navier–Stokes solvers for interface flows use a segregated solution approach, in which the coupled system of equations governing the flow are solved in sequence within a time step. This means that at the point where the interface is to be advected from time *t* to time *t*+Δ*t*, we only have information about the velocity field up to time *t*. But as seen in the integrand in (1.5), calculation of the updated *α*_*i*_ requires information about the velocity field on the interval [*t*,*t*+Δ*t*]. We must therefore estimate the evolution of the velocity field during the time step. The simplest such estimate is to regard the velocity field as constant (in time) during the whole time step. With this assumption, we can write **u**(**x**,*τ*)≈**u**(**x**,*t*) in (1.6). Another assumption we will make in (1.6) is that **u** on the face $F_{j}$ dotted with the differential face normal vector, *d***S**, can be approximated in terms of the volumetric face flux, *ϕ*_*j*_ (defined in (1.9)), as follows:

2.1 $$\text{u}(\text{x},t)\cdot d\text{S}\approx \frac{\phi_{j}(t)}{|{\text{S}}_{j}|}\;dS\;\mathrm{for}\;\text{x}\in F_{j},$$

where *dS*≡*d*|**S**|, and the face normal is given by

2.2 $${\text{S}}_{j}\equiv \int_{F_{j}}\;d\text{S}.$$

Substituting this into (1.6), we obtain

2.3 $$\Delta V_{j}(t,\Delta t)\approx \frac{\phi_{j}(t)}{|{\text{S}}_{j}|}\int_{t}^{t+\Delta t}\int_{F_{j}}H(\text{x},\tau )\;dS\;d\tau .$$

The remaining surface integral in (2.3) is then simply the instantaneous area of face *j* submerged in fluid A, which we will call *A*_*j*_(*τ*):

2.4 $$A_{j}(\tau )\equiv \int_{F_{j}}H(\text{x},\tau )\;dS=\int_{F_{j}\cap A(\tau )}\;dS.$$

Using this definition, we may now write (2.3) as

2.5 $$\Delta V_{j}(t,\Delta t)\approx \frac{\phi_{j}(t)}{|{\text{S}}_{j}|}\int_{t}^{t+\Delta t}A_{j}(\tau )\;d\tau .$$

An important point is that in the special case, where the velocity field is constant in both space and time, (2.5) is exact. Thus, if the cells become sufficiently small compared with the gradients of the velocity field, and the time steps become sufficiently small compared with the temporal variations in the velocity field, the error committed in the above approximation becomes negligible.

As is seen from (2.5), the challenge in constructing a VOF scheme is to estimate the time evolution *within* a time step of the submerged (in fluid A) area of a face, and then integrate this area in time. The time scale on which *A*_*j*_(*τ*) changes is not dictated by the time scales of the flow, but by a complicated combination of the relative orientations of the face and interface, the direction of motion of the interface and the shape of the specific polygonal face. As an example, consider a planar interface approaching a planar face to which it is parallel. In this case, *A*_*j*_(*τ*) will be a discontinuous function of *τ*. This in turn makes Δ*V*_*j*_(*t*,Δ*t*) non-differentiable with respect to Δ*t*. The discontinuous and non-differentiable nature of these quantities is what makes the interface advection problem so difficult to attack with the traditional weaponry of numerical analysis, which relies on the existence of a Taylor expansion of the sought solution.

In the isoAdvector advection step, when we calculate *A*_*j*_(*τ*) for face *j*, our starting point is the isoface in the cell *upwind* of face *j* at time *t*, because this is the cell from which the face receives fluid during the time step. The motion of this isoface *within* the time step [*t*,*t*+Δ*t*] may be approximated by using the velocity data in the surrounding cells. Figure 1*b* shows an example of how the isoface may appear at three times during the time step. For details on our approximation of the isoface motion, the reader is referred to §3.2.

Knowing the isoface position and orientation inside cell *i* at any time within [*t*,*t*+Δ*t*], we also know for its downwind face *j* the face–interface intersection line (see blue lines in figure 1*a*) at any time during the time interval. With this information, the time integral in (2.5) can be calculated analytically, to finally obtain our estimate of the total volume of fluid A transported across face *j* during the time interval [*t*,*t*+Δ*t*].

We stress that the fluid A transport across a face is only calculated once for each face, and that, for internal faces, this value is used to update the volume fractions of *both* of the two cells sharing the face. This guarantees local and global conservation of each of the two fluids A and B.

### 2.3. Algorithm overview

We here give an overview of the steps taken in the isoAdvector algorithm to advance the volume fractions from time *t* to time *t*+Δ*t*:

*Step 1* For each face *j*, initialize Δ*V*_*j*_ with the upwind cell volume fraction, Δ*V*_*j*_=*α*_*upwind*(*j*)_*ϕ*_*j*_Δ*t*.
*Step 2* Find all *surface cells*, i.e. cells with *ϵ*<*α*_*i*_(*t*)<1−*ϵ*, where *ϵ* is a user-specified tolerance (we typically use 10^−8^).
*Step 3* For each surface cell *i*, do the following:
  3.1 Find its isoface, i.e. the isosurface inside the cell with isovalue such that it cuts the cell into the correct volumetric fractions, *α*_*i*_(*t*) and 1−*α*_*i*_(*t*) (details in §3.1).
  3.2 Use the velocity field data to estimate the isoface motion during the time interval [*t*,*t*+Δ*t*] (details in §(b)).
  3.3 For each *downwind* face *j* of surface cell *i*, use the isoface and its motion to calculate the face–interface intersection line during the time interval [*t*,*t*+Δ*t*] (details in §3.1).
  3.4 For each *downwind* face *j* of surface cell *i*, use the motion of its face–interface intersection line to calculate Δ*V*_*j*_(*t*,Δ*t*) from the time integral in (2.5) (details in §3.4).
*Step 4* For each cell calculate *α*_*i*_(*t*+Δ*t*) by inserting the Δ*V*_*j*_s of its faces in (1.7).
*Step 5* For cells with *α*_*i*_(*t*+Δ*t*)<0 or *α*_*i*_(*t*+Δ*t*)>1 adjust the Δ*V*_*j*_s of its faces using a redistribution procedure and recalculate *α*_*i*_(*t*+Δ*t*) by inserting corrected Δ*V*_*j*_s in (1.7). This step also includes an optional subsequent clipping of any cell values *α*_*i*_<0 or *α*_*i*_>1 to ensure strict boundedness before proceeding to the next time step (details in §(e)).

## 3. Implementation details

In this section, we provide the implementation details of the procedure outlined in §2.3. We first note that the time step size may vary between time steps. The user can specify a target *interface* Courant number, Co, based on which time step size is set at the beginning of each time step, to ensure that Co is not exceeded in any surface cell. The interface Courant number only concerns the velocity of the interface normal to itself in surface cells.

Step 1 in §2.3, where we initialize Δ*V*_*j*_ with upwind values, and Step 2, where we find all surface cells with *ϵ*<*α*_*i*_<1−*ϵ*, need no further explanation. We will therefore jump to step 3, which contains the actual calculation of the volume transport across faces.

### 3.1. Calculating the initial isoface in a surface cell

The first step in calculating the isosurface is to interpolate the volume fractions to the grid points of the mesh. The value in a grid point will in general be a linear combination of the volume fractions in the cells sharing this point. Here, we have chosen to use the inverse of the distances between a grid point and the surrounding cell centres as the weights in this interpolation step. Other options could be to use the cell volumes or solid angles as interpolation weights. A systematic study of the influence of this choice is left for future work.

Let us temporarily denote the *N* vertices of cell *i* by **X**_1_,…,**X**_*N*_ and the corresponding interpolated volume fractions by *f*_1_,…,*f*_*N*_. The cell edges are straight lines between pairs of points in the vertex list. To construct the *f*-isoface for cell *i*, we go through all cell edges and cut them by linear interpolation of the edge vertex values: if the edge (**X**_*k*_,**X**_*l*_) has values *f*_*k*_<*f* and *f*<*f*_*l*_, the edge is cut at the point

3.1 $${\text{x}}_{\mathrm{cut}}={\text{X}}_{k}+\frac{f-f_{k}}{f_{l}-f_{k}}({\text{X}}_{l}-{\text{X}}_{k}).$$

Once all such edge cutting points have been found for cell *i*, they can be connected across faces to form the face–interface intersection lines, which can again be connected to form the isoface inside the cell (figure 1*a*). The isoface splits cell *i* into a polyhedral cell, $A_{i}(\;f)$, entirely in fluid A, and another cell, $B_{i}(\;f)$, entirely in fluid B. We can calculate the volume of $A_{i}(\;f)$ relative to the cell volume,

3.2 $$\tilde{\alpha }(\;f)=\frac{\mathrm{vol}(A_{i}(\;f))}{V_{i}}.$$

This will vary monotonically and continuously from 0 to 1, as the isovalue *f* varies from the maximum vertex value, max( *f*_1_,…,*f*_*N*_), to the minimum vertex value, min( *f*_1_,…,*f*_*N*_). As argued in §2.1, the correct isovalue to use is the one recovering the cell volume fraction. That is, we should find f* such that $\tilde{\alpha }(\;f^{*})=\alpha_{i}$. In the current implementation, f* is found by the following procedure. First, we geometrically calculate $\tilde{\alpha }(\;f)$ for the vertex values, *f*_1_,…,*f*_*N*_. Of these, we then find the closest value below and above f*, say, *f*_*k*_ and *f*_*l*_, such that f*∈[*f*_*k*_,*f*_*l*_]. On this interval, we know that $\tilde{\alpha }(\;f)$ varies monotonically like a cubic polynomial. Thus, evaluating $\tilde{\alpha }(\;f)$ geometrically at two points between *f*_*k*_ and *f*_*l*_, we have four equations for the four polynomial coefficients. The resulting linear 4×4 Vandermonde matrix system we solve using LU decomposition. With a polynomial expression for $\tilde{\alpha }(\;f)$ at hand, we can use Newton’s root finding method to efficiently find f* such that $|\tilde{\alpha }(\;f^{*})-\alpha_{i}|<\epsilon$, where *ϵ* is a user-specified tolerance, typically set to *ϵ*=10^−8^. In rare cases, the LU solution does not give useful coefficients because the 4×4 matrix is ill-conditioned, so the method does not converge. In these cases, we use Newton’s root finding method with direct geometric evaluation of $\tilde{\alpha }(\;f)$ instead of the much cheaper polynomial evaluation.

We note that, due to the cell-centre-to-vertex interpolation of the volume fractions, the effective stencil contributing to the isoface inside a surface cell consists of the cell itself with all its point neighbours, that is, all surrounding cells with which it shares a vertex.

### 3.2. Estimating the isoface motion during a time step

We first calculate the geometric face centre, **x**_*S*_, and the unit normal vector, ${\hat{\text{n}}}_{S}$, of the isoface (figure 1*a*). The procedure for doing this is the same as for any other mesh face in OpenFOAM^®^. The average point between the *N* vertex points of the N-gonal face is calculated. Then the face is decomposed into *N* triangles, all sharing this average point as their common apex. The face centre, **x**_*S*_, is then calculated as the area-weighted average of the geometric centres of these *N* triangles. Likewise, the face normal vector, **n**_*S*_, is calculated as the area-weighted average of the *N* triangle area vectors.

The next step is to interpolate the velocity data, **u**_*i*_(*t*), to **x**_*S*_. This is done by first decomposing the cell into tetrahedra, all sharing the cell centre as their common apex. Then we find the tetrahedron containing **x**_*S*_ and interpolate the velocity field into its vertices. Finally, we interpolate linearly from the tetrahedral vertices to obtain the velocity vector **U**_*S*_ at **x**_*S*_. We note that, for stationary meshes, the weightings in this interpolation procedure only need to be calculated and stored once at the beginning of a simulation.

The next step is to dot **U**_*S*_ with the isoface normal, ${\hat{\text{n}}}_{S}$, to obtain the isoface motion normal to itself, $U_{S}\equiv {\text{U}}_{S}\cdot {\hat{\text{n}}}_{S}$. We will make the convention that ${\hat{\text{n}}}_{S}$ is directed from fluid A into fluid B. Thus, positive *U*_*S*_ means that the cell is filling up with fluid A, while negative *U*_*S*_ means that it is filling up with fluid B. In the current implementation, we regard the *U*_*S*_ of an isoface as constant during the whole time step. Possible improvements could be (i) using velocity data from previous time steps to estimate the isoface acceleration during the time step and (ii) calculating the velocity gradient from surrounding cell velocity data to approximate the isoface rotation around its two tangential axes during the time step. Work along these lines is left for future development.

### 3.3. Evolution of the face–interface intersection line

**Figure 2.**
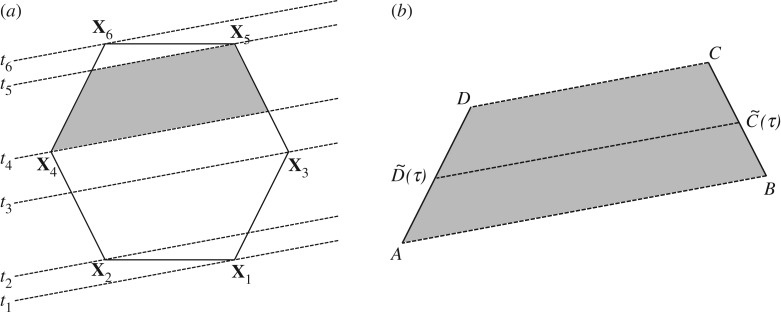
(*a*) Evolving face–interface intersection line drawn for each time it passes a vertex (dashed lines). An example of the area swept between two such times is marked (grey quadrilateral). (*b*) Auxiliary notation for calculation of the face–interface intersection line at intermediate times.

We now use **x**_*S*_, ${\hat{\text{n}}}_{S}$ and *U*_*S*_ to approximate the time evolution of the face–interface intersection line of a face *j*, which is *downwind* of surface cell *i*. This we do by estimating the times at which the isoface, travelling with velocity *U*_*S*_ normal to itself, will reach the vertex points of face *j* (figure 2*a*). Let us temporarily denote the *N* vertex points of face *j* by **X**_1_,…,**X**_*N*_, and the times at which the isoface passes these points by *t*_1_,…,*t*_*N*_. Then we can estimate these times as

3.3 $$t_{k}\approx t+({\text{X}}_{k}-{\text{x}}_{S})\cdot \frac{{\hat{\text{n}}}_{S}}{U_{S}},\;\mathrm{for}\;k=1,\ldots ,N.$$

To obtain the face–interface intersection line at a given time *τ*∈[*t*,*t*+Δ*t*], we can now apply a linear interpolation-based edge-cutting procedure equivalent to the one used to find the initial (i.e. at time *t*) isoface from the volume fractions. Only now the function values at the vertices are the times from (3.3), rather than the interpolated volume fractions.

More specifically, let us temporarily denote by *AB* the line segment at time *t*_*k*_, and by *CD* the line segment at time *t*_*k*+1_, such that *ABCD* is the grey quadrilateral shown in figure 2*a*,*b*. Then, at an intermediate time, *τ*∈[*t*_*k*_,*t*_*k*+1_], we will assume the two endpoints of the face–interface intersection line segment to be

3.4 $$\tilde{D}(\tau )=A+\frac{\tau -t_{k}}{t_{k+1}-t_{k}}(D-A)\;\mathrm{and}\;\tilde{C}(\tau )=B+\frac{\tau -t_{k}}{t_{k+1}-t_{k}}(C-B)$$

as illustrated in figure 2*b*. This concludes our approximation of the face–interface intersection line evolution during a time step.

### 3.4. Time integral of submerged face area

To calculate the time integral of the submerged area, *A*_*j*_(*τ*) in (2.5), we first generate a sorted list of times, ${\tilde{t}}_{1},\ldots {\tilde{t}}_{M}$, starting with ${\tilde{t}}_{1}=t$, and ending with ${\tilde{t}}_{M}=t+\Delta t$, and with a sorted list of all the *t*_*k*_s from (3.3) satisfying *t*<*t*_*k*_<*t*+Δ*t* in between. Then, the time integral in (2.5) can be split up as follows:

3.5 $$\int_{t}^{t+\Delta t}A_{j}(\tau )\;d\tau =\sum_{k=1}^{M-1}\int_{{\tilde{t}}_{k}}^{{\tilde{t}}_{k+1}}A_{j}(\tau )\;d\tau .$$

On each of these subintervals, the face–interface intersection line sweeps a quadrilateral as the one shown in figure 2*b*. Using the definition in (3.4), the submerged area at the intermediate time ${\tilde{t}}_{k}\leq \tau \leq {\tilde{t}}_{k+1}$ is

3.6 $$\begin{array}{rl} A_{j}(\tau ) & =A_{j}({\tilde{t}}_{k})+\frac{1}{2}\;\mathrm{sign}(U_{S})|A\tilde{C}(\tau )\times B\tilde{D}(\tau )|\\ & =P_{k}\tau^{2}+Q_{k}\tau +A_{j}({\tilde{t}}_{k}). \end{array}$$

Here, *P*_*k*_ and *Q*_*k*_ are polynomial coefficients that can be calculated analytically from $A,B,\tilde{C}$ and $\tilde{D}$. The sign of *U*_*S*_ in cell *i* accounts for the direction of propagation of the isoface, i.e. whether the cell and face are gaining or losing fluid A during the time interval. Once these coefficients are obtained, the contribution to the time integral in (3.5) from the sub-time interval $\left[{\tilde{t}}_{k},{\tilde{t}}_{k+1}\right]$ is simply

3.7 $$\int_{{\tilde{t}}_{k}}^{{\tilde{t}}_{k+1}}A_{j}(\tau )\;d\tau =\frac{1}{3}[{\tilde{t}}_{k+1}^{3}-{\tilde{t}}_{k}^{3}]P_{k}+\frac{1}{2}[{\tilde{t}}_{k+1}^{2}-{\tilde{t}}_{k}^{2}]Q_{k}+[{\tilde{t}}_{k+1}-{\tilde{t}}_{k}]A_{j}({\tilde{t}}_{k})$$

Adding up all these sub-interval contributions, as devised by (3.5), and substituting the result into (2.5), we finally reach the sought estimate for Δ*V*_*j*_(*t*,Δ*t*).

As stated in §2.3, the above procedure should be repeated for all downwind faces of a surface cell. On all other faces, we set Δ*V*_*j*_ equal to the volume fraction in their upwind cell multiplied by *ϕ*_*j*_Δ*t*. The updated *α*_*i*_s at time *t*+Δ*t* can now be calculated by inserting the Δ*V*_*j*_s into (1.7).

### 3.5. Bounding procedure

The procedure described above gives an accurate estimate of the fluid transport across faces in many simple cases. It does not, however, guarantee strict boundedness. That is, there is nothing preventing the algorithm from producing updated volume fractions outside the physically meaningful range 0≤*α*_*i*_(*t*+Δ*t*)≤1. Experience shows that slight unboundedness may be produced in cells just behind (i.e. upwind of) the interface. The explanation for this is that while the method’s estimate of the Δ*V*_*j*_s is typically very good, it is not exact. Thus, in cases where a cell is completely emptied or filled during the time step, the small error committed causes the algorithm to miss 0 or 1 by a small amount. If the produced over- and undershoots are sufficiently small, one might be tempted to simply introduce a step in the algorithm that chops *α*_*i*_(*t*+Δ*t*) at 0 and 1 before proceeding to the next time step. However, because this corresponds to removing and adding fluid in cells, this method destroys strict volume conservation and is not true to the VOF idea of only allowing *redistribution* of fluid among cells. While such a step may be practically necessary in order to ensure strict boundedness, it should be used with caution as it may potentially cause severe lack of volume conservation, in particular for long-duration simulations. Can we instead introduce a bounding procedure, which is not adding or removing fluid from the domain, but only redistributes it in order to achieve boundedness? In the following, we will first explain our upper bounding procedure for redistributing the surplus of fluid A in cells with *α*_*i*_(*t*+Δ*t*)>1. Then, we show how the exact same procedure can be used for lower bounding.

#### 3.5.1. Upper bounding

Cells with *α*_*i*_(*t*+Δ*t*)>1 are typically just upwind of the interface, in regions where the interface is moving into fluid B (i.e. *U*_*S*_>0). Therefore, the cells just upwind of an overfilled cell *i* are filled with fluid A, and are therefore not good candidates for taking over the surplus of fluid A in cell *i* in a redistribution step. On the other hand, the cells just downwind of cell *i* are only partially filled with fluid A, and are therefore able to receive cell *i*’s small surplus of fluid A. But if cell *i* has more than one downwind cell, how should its surplus of fluid A be distributed among these? We argue as follows: the overshooting of cell *i* starts at the time t*∈[*t*,*t*+Δ*t*], where the cell becomes filled, i.e. *α*_*i*_(t*)=1. From this time on, all its faces must be completely filled with fluid A. Therefore, pure fluid A will flow through its downwind faces from time t* and onwards. It is therefore natural to pass cell *i*’s surplus of fluid A through its downwind faces using the face fluxes, *ϕ*_*j*_, as the weighting factors. So if the fluid A surplus in cell *i* is *V* ^+^, and the cell has *N* downwind faces with fluxes *ϕ*_1_,…,*ϕ*_*N*_, then the fraction of *V* ^+^ passed on through the *j*’th of these faces should be $\phi_{j}/\sum_{k=1}^{N}\phi_{k}$. However, we will not permit more fluid A to be passed through face *j* than *ϕ*_*j*_(*t*)Δ*t*. Therefore, we will clip the extra flux through face *j* to $\min(\phi_{j}\Delta t-\Delta V_{j},V^{+}\phi_{j}/\sum_{k=1}^{N}\phi_{k})$. If a face reaches its maximum fluid A transport capacity, so the surplus flux is clipped in this way, the result is that not all the surplus *V* ^+^ in cell *i* is passed on to downwind cells in this first redistribution step. In that case, the step is repeated to pass on the remaining surplus of fluid A through the remaining downwind faces, still using the *ϕ*_*j*_s as weightings, and clipping if the maximum capacity of a face is reached. The step is repeated until either all surplus fluid A in cell *i* is passed on to the downwind neighbours, or there are no more downwind cells that can take up more fluid A.

#### 3.5.2. Lower bounding

The procedure for lower bounding (i.e. correcting cells with *α*_*i*_(*t*+Δ*t*)<0) follows simply by changing our perspective from that of fluid A to that of fluid B. We introduce the volume fraction of fluid B, *β*_*i*_≡1−*α*_*i*_, and the volume of fluid B transported across faces during Δ*t*, $\Delta {\tilde{V}}_{j}\equiv \phi_{j}\Delta t-\Delta V_{j}$. Now *α*_*i*_<0 is equivalent to *β*_*i*_>1, and we can apply the upper bounding procedure outlined above to correct the $\Delta {\tilde{V}}_{j}$s. With the $\Delta {\tilde{V}}_{j}$s corrected, we calculate $\Delta V_{j}=\phi_{j}\Delta t-\Delta {\tilde{V}}_{j}$ and insert in (1.7) to obtain the updated volume fraction *α*_*i*_(*t*+Δ*t*).

#### 3.5.3. Clipping

It is our experience that the redistribution process outlined above succeeds in bounding most cells. However, occasionally all downwind faces of an overfilled cell will reach their maximum flux capacity before the cell is fully bounded. This only happens on rare occasions, and when it does it only has a minor effect on the overall quality of the solution. Nevertheless, some applications may require strict boundedness at all times, and so we have to introduce an optional clipping of the volume fractions after the bounding procedure described above and before proceeding to the next time step. When this clipping is switched on the method is not strictly volume-conserving, and one should therefore monitor the evolution of the total volume of fluid A, to ensure that it only varies within acceptable limits.

## 4. Results

In the following, we present the results of simple test cases with isoAdvector. The numerically advected volume fractions should reproduce the solution to an interface advection problem as accurately as possible with the given mesh and time step size. A simple check of this is to advect a confined volume of fluid A across the computational mesh in a uniform velocity field, and observe to what extent the method preserves the shape of the volume as it should.

The other type of test we will perform exploits the time reversibility of the advection problem. If we advect a confined volume of fluid A in a spatially and temporally varying velocity field for a period of time, the interface will be distorted. If we then reverse the flow, and run it backwards for the same amount of time, the volume should return to its initial position and shape.

The following error measures will be used to quantify the solution quality:

— *Shape preservation*. Our quantitative measure of shape preservation will be
  4.1 $$E_{1}(t)\equiv \frac{\sum_{i}V_{i}|\alpha_{i}(t)-\alpha_{i}^{\mathrm{exact}}(t)|}{\sum_{i}V_{i}\alpha_{i}^{\mathrm{exact}}(t)},$$
  where the sums are over all cells and $\alpha_{i}^{\mathrm{exact}}$ is the volume fraction representation of the exact solution at time *t*.
— *Volume conservation*. The relative change in the total volume of fluid A in the domain relative to the initial fluid A volume,
  4.2 $$\delta V_{\mathrm{rel}}(t)\equiv \frac{\sum_{i}\alpha_{i}(t)V_{i}-\sum_{i}\alpha_{i}(0))V_{i}}{\sum_{i}\alpha_{i}(0)V_{i}},$$
  should be zero in simulations, where no fluid A enters or leaves the domain.
— *Boundedness*. For the volume fractions to be physically meaningful, we should have 0≤*α*_*i*_≤1 for *i*=1,…,*N*_*C*_. Our measures of unboundedness will be min_*i*_(*α*_*i*_) and max_*i*_(*α*_*i*_), where the minimum and maximum are taken over all cells at the end of a simulation.
— *Sharpness*. For a sharp interface, the width of the region where *α*_*i*_ changes from 0 to 1 should be similar to the cell size. As the quantitative sharpness measure, we use the volume between the *α*=0.01 and 0.99 isosurfaces of the volume fraction data divided by the corresponding volume for the volume fraction representation of the exact solution. We will call this quantity *δW*_rel_.
— *Efficiency*. Here, we give the simulation times, *T*_calc_, in seconds. All simulations were executed on a single core of an Intel Xeon 3.10 GHz CPU (E5-2687W) on a Dell Precision T7600 Workstation.

For benchmarking the isoAdvector algorithm, we compare its performance with three algebraic VOF schemes, which were all developed for arbitrarily unstructured meshes:

— *MULES*. The interface compression scheme implemented in the OpenFOAM^®^ interfacial flow solver, interFoam. For a good description of this scheme, see Deshpande *et al.* [6]. The scheme does not have a name, but because it uses the Multidimensional Universal Limiter with Explicit Solution (MULES) to keep the VOF data bounded, we will refer to it simply as MULES. All MULES calculations presented in the following were executed using the interFoam solver in OpenFOAM-2.2.0 with the velocity–pressure coupling calculation switched off. As our CFD work is mainly based on OpenFOAM^®^, and our primary aim is to improve its interFoam solver, the main emphasis will be on benchmarking against MULES in the subsequent test cases.
— *HRIC*. The High Resolution Interface Capturing scheme [16], which is, for instance, used in the commercial CFD software STAR-CCM+^®^.
— *CICSAM*. The Compressive Interface Capturing Scheme for Arbitrary Meshes [17], which is, for instance, one among several available options in ANSYS Fluent^®^. In all subsequent CICSAM simulations, we use the recommended blending factor value 0.5. It should be noted that Fluent also has a ‘Geo-Reconstruct’ option, which is a geometric VOF method. We have been unable to find a detailed description of this method in the literature.

For the HRIC and CICSAM calculations, we use our own implementations of the schemes in OpenFOAM^®^. The schemes are available together with the isoAdvector code in the repository [14], where all set-up files for the following test cases may also be found. To also benchmark isoAdvector against geometric VOF schemes, we include for the test case in §4.4 a comparison with error measures listed in the literature for that case.

### 4.1. Disc in steady uniform two-dimensional flow

**Figure 3.**
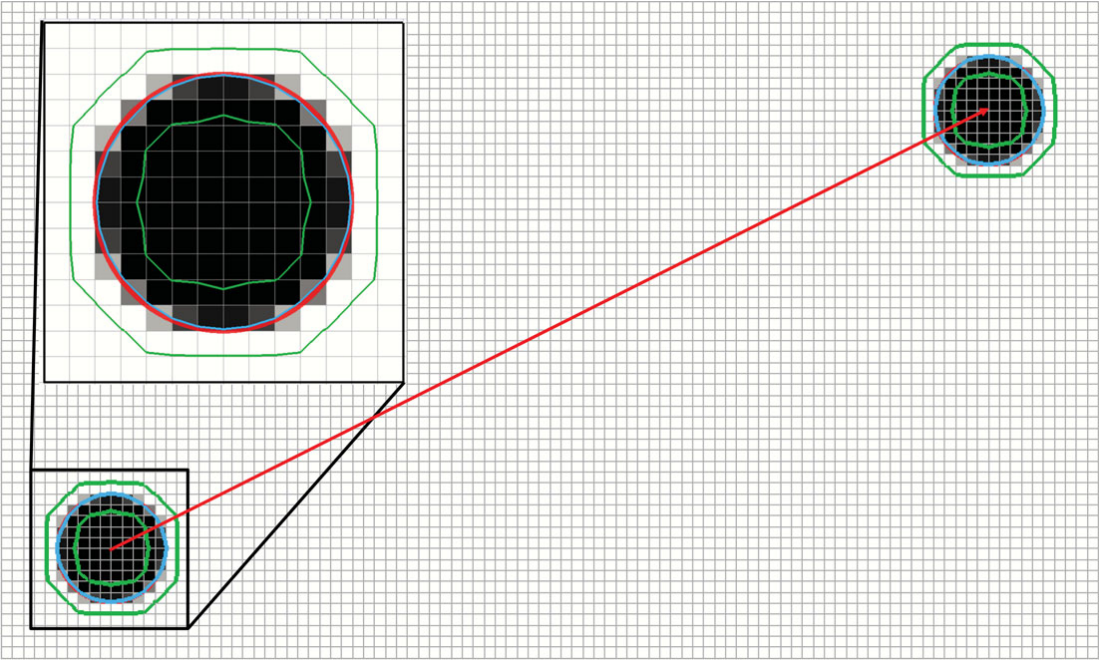
A disc of fluid A of radius 0.25 is initially centred at (0.5,0.5) (lower left corner). It moves with constant velocity **u**=(1,0.5) for 4 s ending at (4.5,2.5) (upper left corner). Volume fractions for the initial and final disc positions are shown with empty cells being white and filled cells being black. Also shown are the *α*=0.5 contour (blue) and the *α*=0.01 and 0.99 contours (green). A zoom of the initial condition is shown in the upper left corner including a red circle marking the exact initial interface.

We start by considering a very simple two-dimensional case on a mesh consisting of square cells: a circular region of fluid A of radius *R*=0.25 moving in a constant and uniform velocity field, **u**=(1,0.5). The initial volume fractions are obtained from the R-isosurface of the function $\sqrt{{\left(x-x_{0}\right)}^{2}+{\left(y-y_{0}\right)}^{2}}$, where (*x*_0_,*y*_0_)=(0.5,0.5) is the initial position of the disc centre. Figure 3 shows the volume fraction representations of the exact initial and final interface in grey scale, with white and black cells meaning empty and filled with fluid A, respectively. The *α*=0.5 contour is shown in blue, and the 0.01 and 0.99 contours are shown in green to indicate the minimal interface width on the given mesh resolution. In the top left corner of figure 3, we also show a zoom on the initial configuration with the exact circle shown in red.

#### 4.1.1. Square meshes

**Figure 4.**
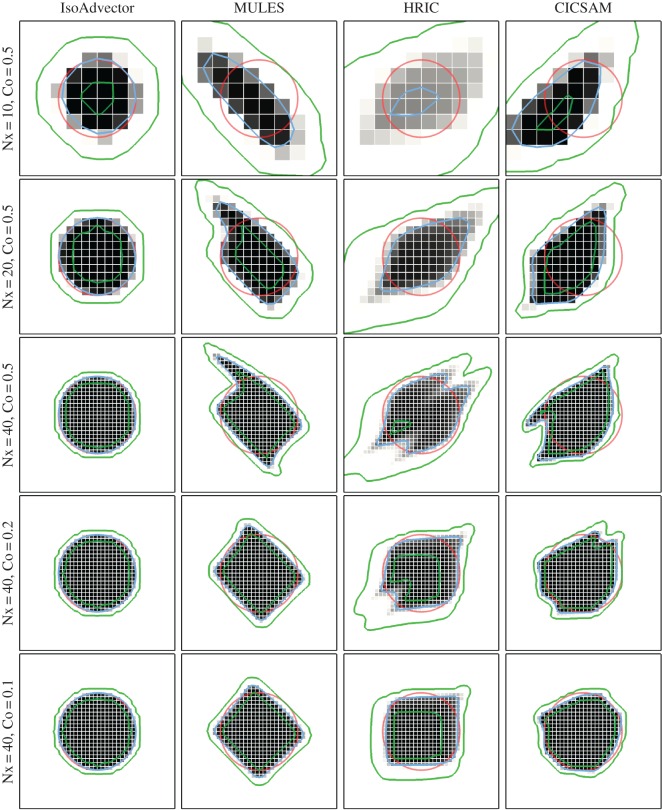
Disc in uniform flow *U*=(1,0.5) at time *t*=4 on a square mesh. Volume fractions shown in grey scale. Exact solution shown with red circles. *α*=0.5 contour shown in blue, and *α*=0.01 and *α*=0.99 contours shown in green.

In figure 4, we show in four columns (left to right) the final volume fraction solutions obtained with isoAdvector, MULES, HRIC and CICSAM with five combinations of mesh and time resolution. In rows 1–3, we investigate the effect of refining the mesh resolution with fixed Courant number, Co=0.5. Then, in row 3–5, we use the finest mesh and reduce Co from 0.5 to 0.2 and 0.1. Error measures and calculation times are displayed in table 1. From figure 4 and table 1, the following observations can be made.

**Table 1.** Performance for disc in uniform flow on a square mesh.

	(Nx,Co)	isoAdvector	MULES	HRIC	CICSAM
(*a*) *E*_1_	(10,0.5)	0.11	0.76	0.86	1
	(20,0.5)	0.035	0.43	0.56	0.73
	(40,0.5)	0.021	0.32	0.38	0.5
	(40,0.2)	0.014	0.2	0.22	0.27
	(40,0.1)	0.017	0.18	0.19	0.15
(*b*) *δV*_rel_	(10,0.5)	0	−0.0019	−0.026	0.0024
	(20,0.5)	−6.7×10^−14^	−0.00018	−0.001	0.0042
	(40,0.5)	−3.6×10^−10^	−1.1×10^−5^	−1×10^−5^	0.0012
	(40,0.2)	−4.7×10^−13^	−5.2×10^−10^	1.5×10^−5^	0.00044
	(40,0.1)	−2.1×10^−13^	−2.7×10^−13^	−8.8×10^−5^	0.00019
(*c*) $\min_{i}(\alpha_{i})$	(10,0.5)	−3.9×10^−15^	0	0	−0.084
	(20,0.5)	0	0	0	−0.24
	(40,0.5)	0	0	−5.9×10^−13^	−0.23
	(40,0.2)	0	0	0	−0.091
	(40,0.1)	0	0	0	−0.047
(*d*) $1-\max_{i}(\alpha_{i})$	(10,0.5)	0	0.024	0.46	−0.075
	(20,0.5)	0	3.4×10^−11^	0.063	−0.16
	(40,0.5)	0	0	0.0069	−0.18
	(40,0.2)	0	4×10^−14^	0.00035	−0.1
	(40,0.1)	5×10^−14^	6.4×10^−13^	2.1×10^−5^	−0.034
(*e*) *δW*_rel_	(10,0.5)	−0.0003	0.34	1.4	0.21
	(20,0.5)	−0.0051	0.44	1.9	0.2
	(40,0.5)	0.026	0.51	3	0.27
	(40,0.2)	0.012	0.32	1.7	0.14
	(40,0.1)	0.0065	0.35	1.2	0.036
( *f*) *T*_calc_	(10,0.5)	0.22	0.52	0.22	0.19
	(20,0.5)	0.85	2.16	0.94	0.87
	(40,0.5)	4.27	12.42	5.28	4.49
	(40,0.2)	7.41	28.28	9.46	8.4
	(40,0.1)	12.94	55.13	16.82	14.61

*Shape preservation*. The visual impression from figure 4 is that isoAdvector is superior at preserving the shape of the disc on all shown mesh-Courant number combinations. MULES has a tendency to align the interface at 45 degrees with the mesh faces. Therefore, the MULES solution converges to a tilted square shape as cell and time step sizes are refined (2nd column in figure 4). The HRIC scheme shows a tendency to align the interface with the mesh faces, as also reported in Nielsen [18]. This causes the initially circular interface to converge to a square (3rd column in figure 4). For all the Co=0.5 runs (4th column, rows 1–3 in figure 4), CICSAM does not perform very well in terms of shape preservation. However, it is the only one of the reference schemes which converges to something resembling a circular interface solution as the time step is decreased (lower right corner in figure 4). Table 1*a* quantifies these observations, showing that the isoAdvector *E*_1_ error is at least a factor of 7 smaller than the best of the other schemes for all runs. The table also reveals that the isoAdvector solution only improves slightly, when going from Co=0.5 to Co=0.2, and becomes slightly worse from Co=0.2 to 0.1. Increasing errors with decreasing time step size on a fixed mesh was also reported in Ubbink & Issa [17]. From the three Co=0.5 errors in table 1*a*, we calculate isoAdvector’s order of convergence with mesh refinement to be ∼2.4.

*Volume conservation*. From table 1*b*, we see that isoAdvector is the only scheme with volume preservation down to machine precision even on the coarsest mesh. On the finest mesh MULES also performs very well, followed by HRIC. CICSAM is the worst performing scheme in this comparison.

*Boundedness*. From tables 1*c* and 1*d*, we see that isoAdvector keeps the volume fraction data bounded to within machine precision. Also MULES and HRIC produce bounded volume fractions, whereas CICSAM has severe bounding problems even on the finest mesh.

*Sharpness*. Table 1e shows our sharpness measure, *δW*_rel_. For all simulations, the isoAdvector thickness is very close to the best one can expect, i.e. the thickness of the volume fraction representation of the exact solution on the given mesh. The MULES interface width is only 30–50% larger than the width of the exact solution. HRIC performs rather badly in terms of interface sharpness with a smearing of the interface which is clearly visible in figure 4 (column 3). CICSAM keeps the interface sharp for all runs and is the best performing of the reference schemes in this respect.

*Efficiency*. From table 1*f*, we see that, for this simple test case, isoAdvector is slightly faster than the fastest reference schemes, CICSAM and HRIC, for most simulations, and two to four times faster than MULES. It is remarkable that the isoAdvector scheme can obtain a significantly improved accuracy with this significantly lower usage of computer resources.

#### 4.1.2. Unstructured meshes

**Figure 5.**
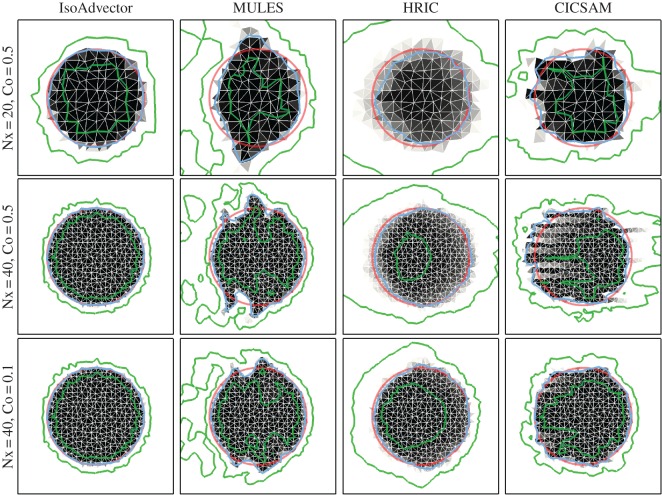
Disc in uniform flow *U*=(1,0) at time *t*=4 on a triangle mesh.

**Figure 6.**
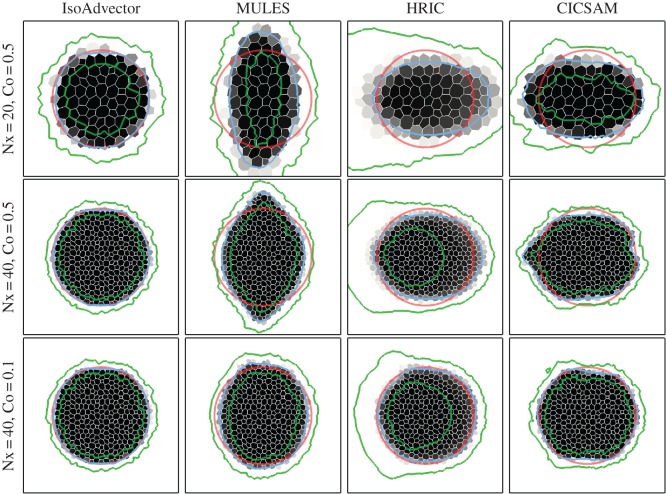
Disc in uniform flow *U*=(1,0) at time *t*=4 on a polygon mesh.

In figures 5 and 6, we show a sequence of simulations similar to those in figure 4, but now on triangular and polygonal meshes, respectively. Again the columns show ( from left to right) the solutions obtained with isoAdvector, MULES, HRIC and CICSAM. From row 1 to 2 we refined the mesh, keeping the Courant number at =0.5. From row 2 to 3 we retain the mesh, but go from Co=0.5 to 0.1. As the meshes have no preferred direction, we use velocity **u**=(1,0) for these simulations. The disc radius is still *R*=0.25, and the solutions are shown at time *t*=4. Inspection of figures 5 and 6 and the quantitative measures (here only *E*_1_ is shown in tables 2 and 3) reveals that most of the observations listed above for the square mesh also hold for the triangle and polygon meshes. There are, however, a number of differences concerning the performance of the reference schemes.

**Table 2.** *E*_1_ for simulations in figure 5. Nx in the left column is the resolution of the square base mesh from which the unstructured meshes were generated by randomly distorting the points followed by a Delaunay triangulation.

(Nx,Co)	isoAdvector	MULES	HRIC	CICSAM
(20,0.5)	0.029	0.27	0.43	0.23
(40,0.5)	0.014	0.18	0.26	0.27
(40,0.1)	0.014	0.13	0.15	0.086

**Table 3.** *E*_1_ for simulations in figure 6. Meshes are dual meshes of triangle meshes in figure 5.

(Nx,Co)	isoAdvector	MULES	HRIC	CICSAM
(20,0.5)	0.039	0.43	0.45	0.32
(40,0.5)	0.02	0.25	0.23	0.22
(40,0.1)	0.024	0.13	0.14	0.099

First, the tendency of MULES to align the interface at 45 degrees with the mesh faces is no longer visible due to the random face orientations, which presumably causes this systematic error to cancel out. However, on the triangle mesh, MULES still does not seem to converge to a circular interface due to the development of ‘wings’ on the sides (relative to the flow direction) of the fluid A region. On the polygon mesh, MULES does significantly better in terms of shape preservation, though with a tendency to squeeze the interface along the direction of motion.

Second, HRIC is much better at preserving the interface shape on both the triangle and polygon mesh than on the square mesh. It is, however, still very diffusive. This is a good example of a case where the *α*=0.5 contour (blue) alone would give an impression of good performance, but where the *α*=0.01 and 0.99 contours (green) reveal the excessive smearing of the interface.

Third, CICSAM performs very poorly on the triangle mesh with threads of fluid B piercing into the disc volume from behind. On the polygon mesh, these threads are not present and the solution quality is similar to the square mesh solution. On both the triangle and the polygon mesh, CICSAM has the same problems with unboundedness that we saw on the square mesh.

We conclude that also on unstructured meshes in two-dimensional the performance of isoAdvector is significantly better than the reference scheme with calculation times that are similar to HRIC and CICSAM, and significantly lower than MULES.

### 4.2. Spiralling disc

**Figure 7.**
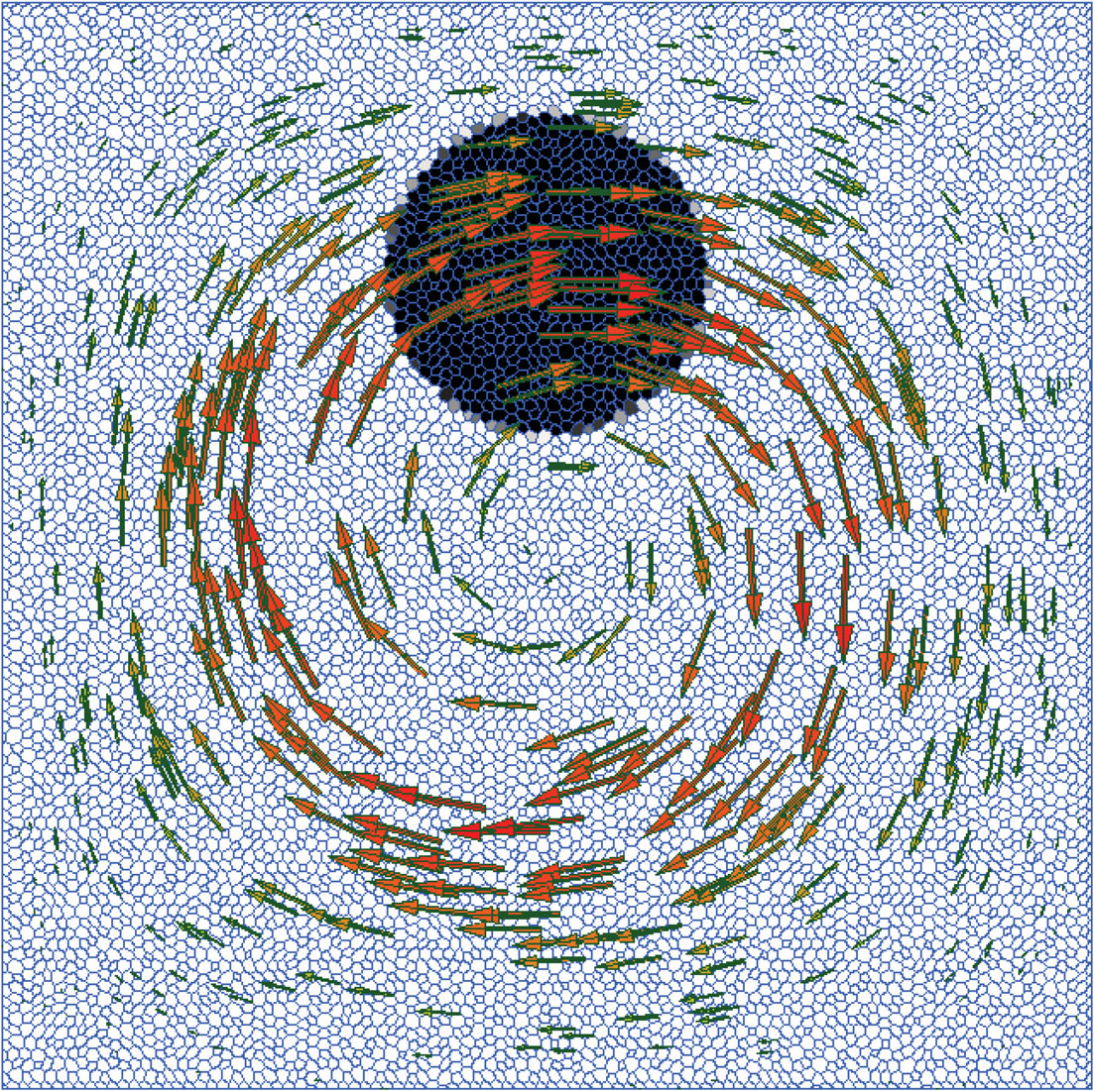
Initial condition for the spiralling disc test case.

After two-dimensional uniform flow tests, our next step is to test the solver performance in a spatially varying flow. We adopt the set-up shown in figure 7, which has become a standard case for testing the ability of an interface advection schemes to deal with severe interface stretching [17,19,20–25]. The domain is the unit square with a disc of radius *R*=0.15 initially placed at (*x*,*y*)=(0.5,0.75). The velocity field is given by

4.3 $$\text{u}(x,y,t)=\cos\left(\frac{2\pi t}{T}\right)(-\sin^{2}(\pi x)\sin(2\pi y),\;\sin(2\pi x)\sin^{2}(\pi y)),$$

where the period of the flow is set to *T*=16. This flow stretches the disc into a long filament until, at time *t*=4, the flow is completely attenuated by the temporally varying cosine prefactor. Then, the flow reverses and the volume of fluid flows back into its original shape at time *t*=8. At this time, our shape preservation error measure, *E*_1_, can be calculated by comparing the computed final state with the initial state. As we know the flow in advance, we use the fixed intermediate velocity, **u**(*x*,*y*,*t*+0.5Δ*t*), on the whole time interval [*t*,*t*+Δ*t*].

**Figure 8.**
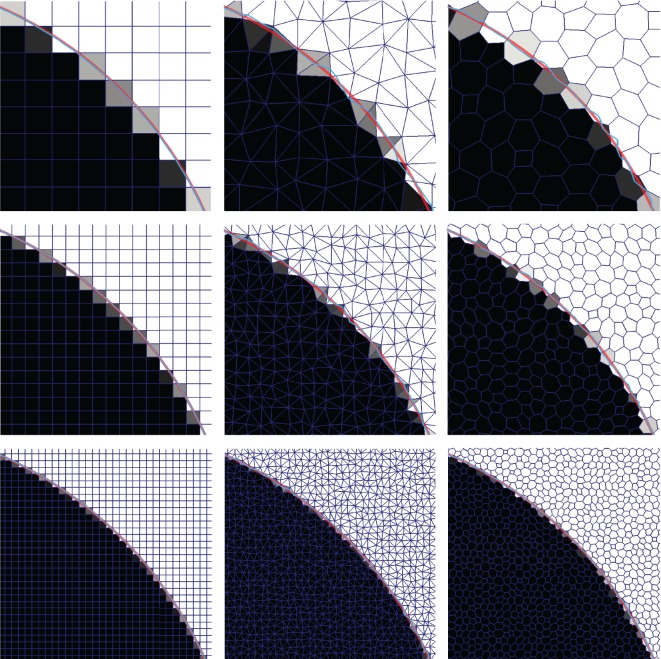
Meshes used to study the spiralling disc case. Zoom on part of the initial interface. Exact circular shape shown in red and the 0.5-contour of volume fractions shown in blue.

**Figure 9.**
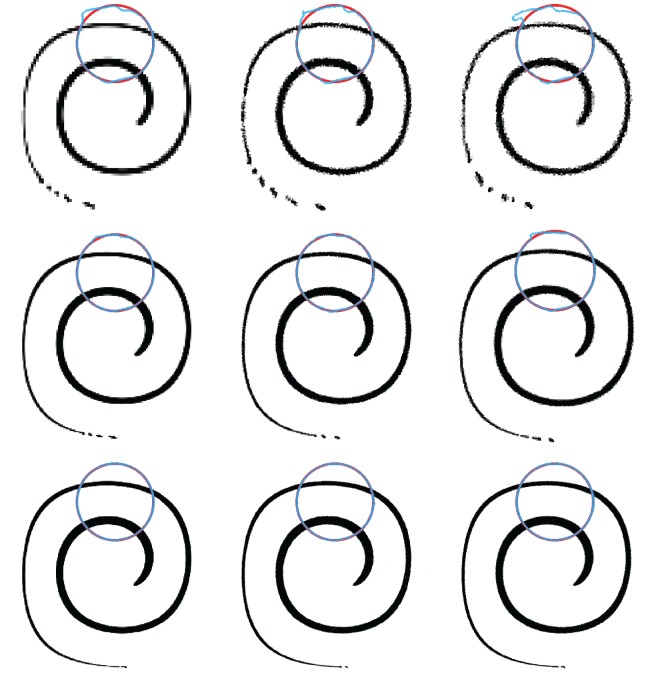
Spiraldisc isoAdvector results with Co=0.5 on meshes from figure 8.

In figure 8, we show the square, triangle and polygon meshes in three different resolutions on which the isoAdvector method was tested. The results are shown in figure 9 using the same arrangement of the meshes. All simulations are run with Co=0.5. In each panel, the exact initial and final interface shape is shown with a red circle overlaid with the *α*=0.5 contour (blue) of the final (i.e. at time *t*=8) volume fraction data. The spiral-shaped volume of fluid at time *t*=4, where it is maximally stretched, is also shown in each panel.

All simulations show some degree of pinching at *t*=4. This occurs when the filament thickness reaches the cell size as is to be expected. The phenomenon is therefore most pronounced on the coarsest meshes. We note that although the exact mathematical solution does not pinch, the 0.5-contour of its volume fraction representation *will* indeed pinch if the mesh is coarse enough. As such, pinching does not have to be an error. However, as droplets pinch off, and the local interface curvature becomes comparable with the cell size, the isofaces are not able to represent the significant interface curvature inside a cell. The isoface-based approximation of the advection then becomes faulty, leading to errors in the estimate of the droplet motion similar to those reported in Cerne *et al.* [26]. The irreversibility of the introduced errors causes a distortion of the final disc in its upper region, which is made up of the previously pinched-off fluid.

The mesh sizes, error measures and calculation times are shown in table 4. From the *E*_1_ values in table 4, the orders of convergence with mesh refinement are calculated to be 1.9, 1.7 and 1.9 for the square, triangle and polygon meshes, respectively.

**Table 4.** Spiralling disc isoAdvector simulations with Co=0.5.

mesh		square	triangle	polygon
(*a*) #cells	1	10 000	19 602	10 000
	2	40 000	79 202	40 000
	3	160 000	318 402	160 000
(*b*) *E*_1_	1	0.047	0.054	0.071
	2	0.012	0.02	0.018
	3	0.0023	0.0095	0.0039
(*c*) *δV*_rel_	1	−1.5×10^−14^	−5.9×10^−15^	−5×10^−15^
	2	3.4×10^−14^	−9.6×10^−14^	−1.8×10^−14^
	3	2.1×10^−14^	−4.6×10^−13^	−1.8×10^−13^
(*d*) min_*i*_(*α*_*i*_)	1	−6.1×10^−8^	−7.2×10^−9^	0
	2	−2.8×10^−7^	−1.6×10^−10^	0
	3	−4.7×10^−7^	−8.4×10^−12^	0
(*e*) 1−*max*_*i*_(*α*_*i*_)	1	−5.1×10^−8^	0	0
	2	−1.8×10^−8^	0	0
	3	−1.4×10^−8^	0	0
( *f*) *T*_calc_	1	13	58	26
	2	60	304	133
	3	314	1815	718

**Figure 10.**
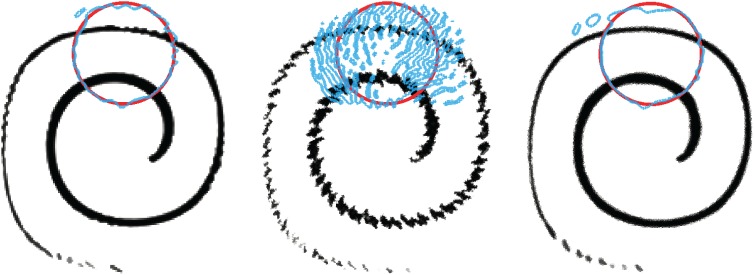
Spiraldisc simulation MULES results with Co=0.1 on the three intermediate resolution meshes from figure 8.

For a comparison, we show in figure 10 and table 5 the results obtained with MULES on the intermediate resolution meshes of the three types, using Co=0.1. For the square mesh, the MULES *E*_1_ error is ∼50% larger than the corresponding isoAdvector error. For the triangle mesh, the final interface is completely disintegrated. On the polygon mesh, MULES also gives acceptable results, although the *E*_1_ error is five times larger than the isoAdvector error on the same mesh. In terms of calculation times, MULES is ∼10 times slower than isoAdvector. This is in part because MULES is run with smaller time steps. However, we also ran the simulations with Co=0.5, in which case the MULES results on all three meshes were completely disintegrated like the triangle mesh solution in figure 10.

**Table 5.** Spiralling disc error measures for MULES with Co=0.1 on intermediate meshes (to compare with middle row in figure 9 and meshes 2 in table 4).

mesh	square	triangle	polygon
*E*_1_	0.072	0.66	0.09
*δV*_rel_	2.8×10^−14^	−3.1×10^−14^	−2×10^−15^
*T*_calc_	553	4151	1355

### 4.3. Sphere in steady uniform three-dimensional flow

**Figure 11.**
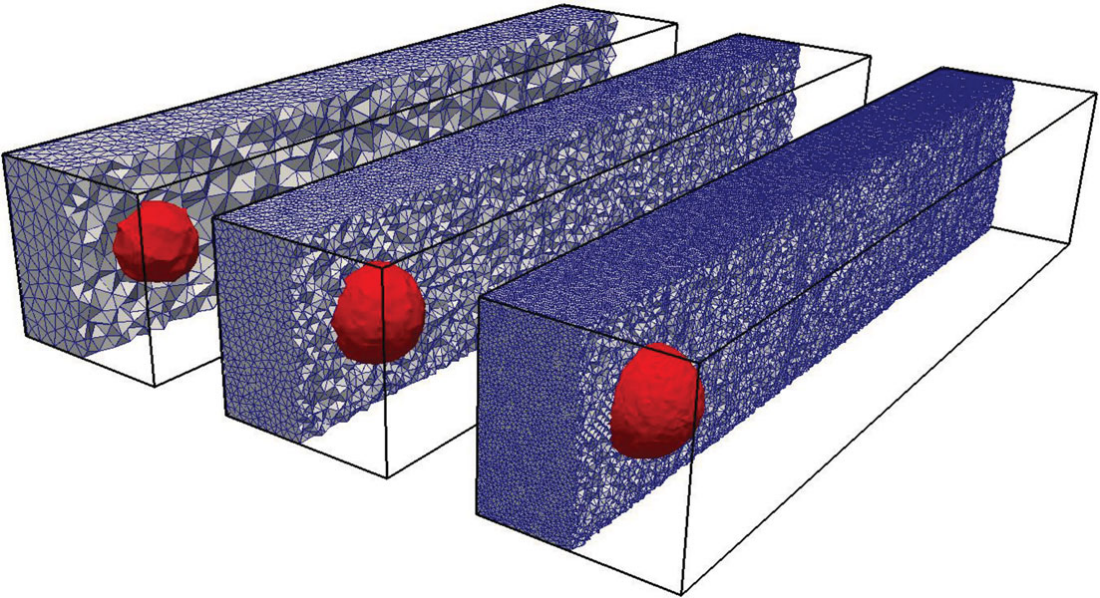
Random tetrahedron meshes used for a sphere in the uniform flow test case. The *α*=0.5 isosurface shown for initial volume fraction data.

**Figure 12.**
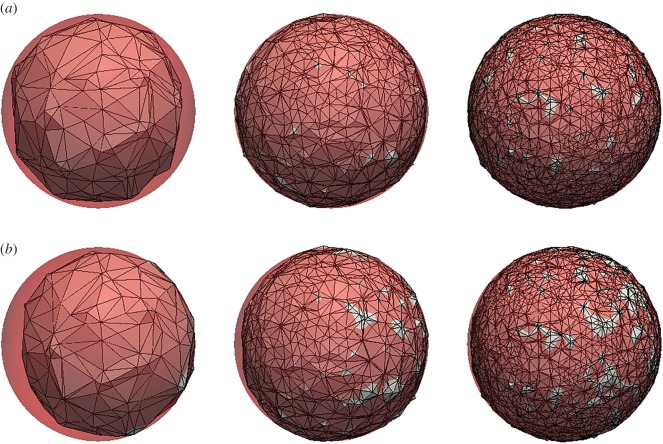
Sphere in uniform flow *U*=(0,0,1) on tetrahedral mesh at time *t*= 4. (*a*) Exact solution (red sphere) and its 0.5-isosurface on three different mesh resolutions. (*b*) Exact solution (red sphere) and the 0.5-isosurface of the isoAdvector solution with Co=0.5.

In this test, we go back to a uniform flow, but now in three dimensions. The velocity is *U*=(0,0,1), and the initial interface is a sphere of radius *R*=0.25 centred at (0.5,0.5,0.5). The simulations are run on three meshes consisting of 49 868, 343 441 and 1 753 352 random tetrahedral cells covering the domain, [0,1]×[0,1]×[0,5]. The meshes and the 0.5-isosurface of the initial volume fraction data are shown in figure 11. The simulations are run with Co=0.5 until *t*=4, where the sphere has moved to (0.5,0.5,4.5). The results are shown in figure 12 and table 6. In figure 12*a*, we show the exact final sphere (red) and the 0.5-isosurface of its volume fraction representation on the three mesh resolutions. In figure 12*b*, we show the exact sphere (red) together with the 0.5-isosurface of the final volume fraction data obtained with isoAdvector. As seen from table 6, the *E*_1_ error on the coarsest mesh is fairly large. From figure 12 (lower left panel), we see that this lack of overlap is mainly due to an overestimation of the propagation speed rather than a lack of ability to retain the spherical interface shape. On the finer meshes *E*_1_ is reduced significantly, although the tendency to be slightly ahead of the exact solution is still visible in figure 12. The linear cell size is reduced by a factor of 1.9 from the coarse to intermediate mesh, and by a factor of 1.7 from the intermediate to fine. Based on these ratios and the *E*_1_s in table 6, the convergence order is calculated to lie in the range 2.6–3.2.

**Table 6.** Sphere in uniform flow errors and calculation times for isoAdvector with Co=0.5.

*n* cells	*E*_1_	*δV*_rel_	$\min_{i}(\alpha_{i})$	$1-\max_{i}(\alpha_{i})$	*δW*_rel_	*T*_calc_
49 868	0.18	−2.8×10^−11^	0	0	−0.033	11
343 441	0.046	−7.8×10^−12^	−6.9×10^−11^	0	0.0067	157
1 753 352	0.021	5.9×10^−11^	−2.7×10^−9^	0	0.0035	1411

**Figure 13.**
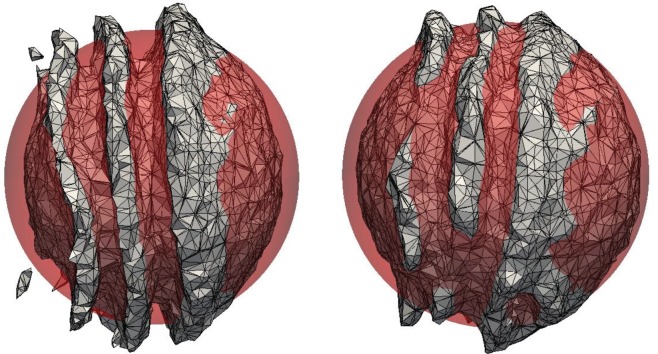
Sphere in uniform flow *U*=(0,0,1) at time *t*=4 on the finest tetrahedral mesh of figure 12. (*a*) Exact solution (red sphere) and 0.5-isosurface of MULES solution with Co=0.5. (*b*) The same but with Co=0.1.

For comparison, we show in figure 13 and table 7 the results obtained with MULES on the finest mesh running with Co=0.1 and 0.5. In both cases the shape preservation is significantly worse than the isoAdvector results. It is also notable that the MULES simulations with Co=0.1 and Co=0.5 are, respectively, 20 and 5 times slower than the corresponding isoAdvector simulation with Co=0.5.

**Table 7.** Sphere in uniform flow error measures for MULES on the finest tetrahedron mesh.

Co	*E*_1_	*δV*_rel_	$\min_{i}(\alpha_{i})$	$1-\max_{i}(\alpha_{i})$	*δW*_rel_	*T*_calc_
0.5	0.42	−4.5×10^−13^	−5×10^−6^	−1.9×10^−6^	2	7306
0.1	0.29	−8.2×10^−13^	0	9×10^−6^	1.9	28 686

### 4.4. Sphere in non-uniform three-dimensional flow

**Figure 14.**
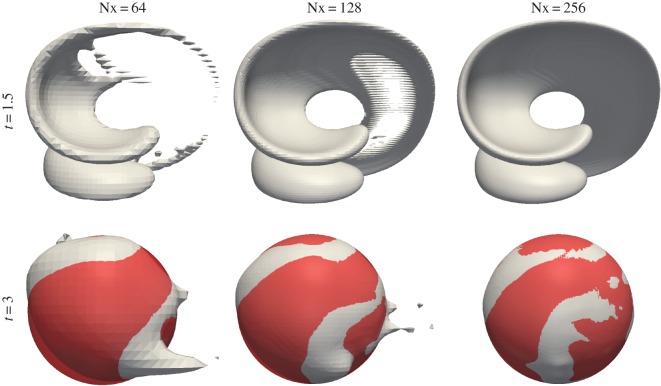
*α*=0.5 isosurfaces for sphere in non-uniform flow with Co=0.5. Results for three mesh resolutions are shown at the time of maximum strechting, *t*=1.5, and at the final time, *t*=3. Exact final solution is shown with red spheres.

Our final test case is also in three dimensions, but now with a non-uniform velocity field. We adopt a configuration from LeVeque [27], which has become a standard test case [19,28–30] for testing interface advection methods and their ability to deal with highly distorted interfaces in three dimensions. The domain is the unit box, and the initial interface is a sphere of radius *R*=0.15 centred at (0.35,0.35,0.35). This surface is advected in the velocity field,

4.4 $$\text{u}(x,y,z,t)=\cos\left(\frac{2\pi t}{T}\right)\left(\begin{array}{c} 2\sin^{2}(\pi x)\sin(2\pi y)\sin(2\pi z)\\ -\sin(2\pi x)\sin^{2}(\pi y)\sin(2\pi z)\\ -\sin(2\pi x)\sin(2\pi y)\sin^{2}(\pi z) \end{array}\right),$$

where the period is set to *T*=6. This flow stretches the sphere into a thin sheet creating two bending and spiralling ‘tongues’. The maximum deformation is reached at *t*=1.5, where the temporal cosine prefactor completely quenches the flow. From here on the flow reverses, and the interface returns to its initial shape and position at time *t*=3. In figure 14, the isoAdvector results are shown at time *t*=1.5 in the top row, and at time *t*=3 in the bottom row, on three cubic meshes with $dx=\frac{1}{64},\frac{1}{128}$ and $\frac{1}{256}$. In the lower panels, the exact final spherical shape is also shown in red. From ODE calculations with the velocity field (4.4), we have measured the sheet thickness at *t*=1.5 to be ∼0.0063. This, and the fact that an edge can at most be cut once by the prescribed isosurface routine, explains why there are holes in the 0.5-isosurface of the volume fraction data on the two coarsest meshes with d*x*≈0.016 and d*x*≈0.0078, and no holes in the finest simulation with d*x*≈0.0039. The error measures and calculation times for the three simulations are shown in table 8. Based on the *E*_1_s in this table, the order of convergence is calculated to be 2.2.

**Table 8.** Error measures and calculation times for isoAdvector simulations in figure 14 of a sphere in a three-dimensional non-uniform flow on three cube meshes.

Nx	*E*_1_	*δV*_rel_	$\min_{i}(\alpha_{i})$	$1-\max_{i}(\alpha_{i})$	*δW*_rel_	*T*_calc_
64	0.21	−1.5×10^−13^	−2.6×10^−10^	0	0.42	110
128	0.045	1.2×10^−12^	−1.1×10^−7^	0	0.087	1260
256	0.0086	−9.4×10^−9^	−1.1×10^−10^	0	0.0044	25 988

Error measures for this test case for three different geometric VOF approaches are listed in table 7 of Liovic [29]. The error is given as the *L*_1_-norm, which is related to the *E*_1_ measure by *L*_1_=*E*_1_*V*_*A*_, where *V*_*A*_=4/3*πR*^3^≈0.0141 is the volume of fluid A in the domain. Thus, the isoAdvector *E*_1_ values in table 8 for the (64,128,256) meshes translate into *L*_1_=(3.0×10^−3^,6.4×10^−4^,1.2×10^−4^). Comparison with table 7 in Liovic [29] shows that all listed errors are similar in magnitude, with the exception that, on the finest mesh, the error of the method denoted by ‘CVTNA+PCFSC unsplit’ in Liovic [29] is approximately three times smaller than the other approaches in that paper and almost twice as small as our error. We note that the schemes described in Liovic [29] are developed for orthogonal hexahedral meshes. Our error measures are also similar in magnitude to those presented in table IV of Xie *et al.* [12], which shows the *L*_1_-norm for their method (developed for unstructured meshes consisting of hexahedra), and those described in Hernandéz *et al.* [8] (hexahedral meshes) and Jofre *et al.* [13] (general meshes).

**Figure 15.**
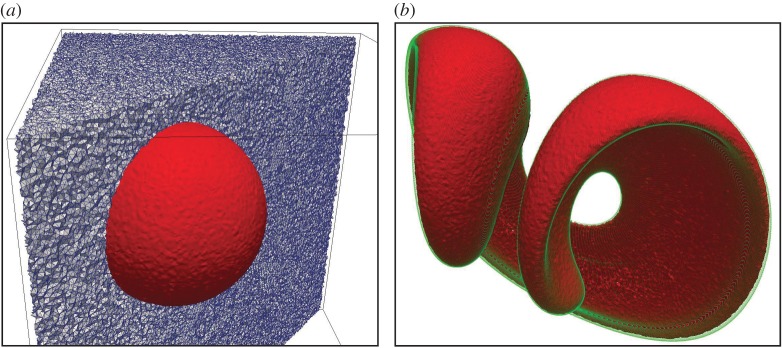
Sphere in non-uniform flow on the tetrahedral mesh. (*a*) Mesh and the 0.5-isosurface of the initial volume fraction data. (*b*) isoAdvector solution (red) and the solution obtained with an accurate ODE solver (green) at time *t*=1.5.

As a final test, we have repeated this simulation on a mesh consisting of random tetrahedra. To get sufficient resolution to avoid holes in the 0.5-isosurface of the solution, we used a mesh with 10 131 041 cells covering [0.15,0.90]×[0.15,0.80]×[0.15,0.80]. A cut through this mesh and the 0.5-isosurface of the volume fraction representation of the initial sphere are shown in figure 15*a*. In figure 15*b*, we show the isoAdvector solution at time *t*=1.5, where the stretching is maximal. This panel also contains a solution obtained by integrating the velocity field with a Runge–Kutta ODE solver for 160.000 points evenly distributed on the initial sphere (green dots). The visual impression from figure 15 is that there is a good match between the ODE and the isoAdvector solutions. At time *t*=3, the shape was preserved with an error of *E*_1_=0.014. Owing to the clipping procedure which was activated in the bounding step, *δV*_rel_ was −0.63%. Bounding errors were in the order of 10^−5^. The simulation took 67 h on a single core and 23 h on eight cores. As the parallelization of the code is based on domain decomposition, the scalability depends on the extent to which surface cells are evenly distributed among the processors.

## 5. Conclusion

We have developed a new algorithm, isoAdvector, for numerical interface advection across general structured and unstructured computational meshes. The method is derived from ‘first principles’, i.e. from the control volume integrated continuity equation for a discontinuous density field. The isoAdvector scheme belongs to the class of geometric VOF methods, but with novel ideas implemented in both the interface reconstruction step and in the interface advection step.

The novelty in the reconstruction step is the usage of efficient isosurface calculations to estimate the distribution of fluids inside computational cells. This is a very robust method even on unstructured meshes. It avoids the gradient calculations traditionally used in the geometric VOF reconstruction step, which may cause problems, because the numerically estimated gradient is a cell volume averaged Dirac *δ*-function.

In the advection step, the approach taken in existing geometric VOF methods for general meshes is to calculate so-called flux polyhedra and their intersection with the submerged part of the mesh cells [10,11,13]. This is an expensive and complicated procedure, which we avoided in our work. Instead, we focus on the time evolution of the submerged part of a face during a time step. We approximate the face–interface intersection as a line sweeping the face during the time step, and divide the time step into sub-time intervals defined by the times at which the line passes the face vertices. On such time intervals, we can then analytically calculate the volume of fluid A passing a face under the assumption that the line is moving steadily across the face during the interval. In the development of this procedure, no assumptions are made on the shape of a face, and therefore also the advection step is by design applicable on general meshes.

We have given a proof-of-concept by testing the isoAdvector method on various simple flow–interface combinations both in two-dimensional and three-dimensional structured and unstructured meshes. The results are very satisfactory both in terms of shape preservation, volume conservation, boundedness, interface sharpness and efficiency. The order of convergence with mesh refinement varies between 1.7 and 3.2 for the test cases presented here. Also, in spite of the geometric nature of some of the steps involved, the implementation of the new algorithm is relatively straightforward.

The isoAdvector advection step relies on local geometric considerations based on available information from a surface cell and its nearest neighbours. Thus, while there seems to be no strict limit of Co<1, the underlying geometric considerations *will* become questionable if the time step is so large that the interface moves across many cells during one time step. We therefore do not expect the method to perform well with interface Courant numbers significantly higher than 1. For Co in the range [0,1], it is our experience that the solution quality is fairly stable, albeit with an optimum around 0.5. This is to be contrasted with the explicit MULES scheme in OpenFOAM^®^’s interFoam solver, which in our experience is limited to Co≤0.1 if accuracy is important.^2^

We note that because the governing equation we solve is the passive advection equation for a scalar field in a solenoidal velocity field, the isoAdvector method may also find applications within other branches of CFD, where the advected surface is not necessarily marking the interface between two distinct fluids. There are many situations where one needs to follow a passive tracer field, e.g. representing the concentration of some substance, which is immiscible with the surrounding fluid. Another possible application could be in an immersed boundary method, where the isoAdvector scheme could provide accurate estimates of the fluid–solid interface within computational cells.

Based on the interFoam solver in OpenFOAM^®^, we are currently working on a consistent coupling of isoAdvector with a pressure–velocity solver. The performance of the resulting new interfacial flow solver will be presented in a future paper. Finally, we note that due to its applicability on arbitrary meshes, the isoAdvector code can be coupled with an adaptive mesh refinement routine with only minor modifications. Such a coupling will also be investigated in future work.

The isoAdvector code is published [14] as an open source extension to OpenFOAM^®^. The code has been parallelized based on the domain decomposition approach of OpenFOAM^®^, and we are currently modifying the code to work also on moving meshes. It is our hope that the isoAdvector concept and code will be used, tested and further developed by the CFD community, and eventually result in improved simulation quality in the broad field of applications involving interfacial flows.

## Footnotes

1 On the surface S one could set $\rho =\frac{1}{2}(\rho_{A}+\rho_{B})$ for *ρ* to be defined everywhere. However, as S has zero volume, the value of *ρ* on S is immaterial.

2 It should be mentioned that, from OpenFOAM^®^ v. 2.3.0, a new semi-implicit MULES scheme is introduced to solve some of the issues with boundedness, stability and accuracy for large Courant numbers. The literature documenting the ideas behind and the performance of this new method is, however, still very sparse.

## Data accessibility

The isoAdvector code is publicly available in the repository www.github.com/isoadvector together with the set-up files necessary to reproduce the results of all test cases included in this paper. The code is an extension to the open source C++ code library, OpenFOAM, which can be downloaded from www.openfoam.com. The unstructured meshes used in this paper are available in the OpenFOAM polyMesh format from the Dryad Digital Repository: http://dx.doi.org/10.5061/dryad.66840 [31].
